# Study on Porosity of Thermal-Sprayed Commercially Pure Aluminum Coating

**DOI:** 10.3390/ma16196612

**Published:** 2023-10-09

**Authors:** Bo Li, Lei Fan, Jie Bai, Jinhang He, Jianfeng Su, Song Wang, Chao Deng, Shifeng Liu, Zhiqing Zhang

**Affiliations:** 1 Electric Power Research Institute, Guizhou Power Grid Co., Ltd., Guiyang 550000, China; gzgylb2207@163.com (B.L.); flgkl2009@126.com (L.F.);; 2 An Shun Power Supply Bureau, Guizhou Power Grid Co., Ltd., Anshun 561000, China; sujianfeng02@126.com; 3 College of Materials Science and Engineering, Chongqing University, Chongqing 400044, Chinaliusf06@cqu.edu.cn (S.L.);

**Keywords:** coating, mechanical polishing, argon ion beam, electrolytic polishing, porosity

## Abstract

Porosity is closely related to the corrosion and wear properties of a coating processed by thermal-spraying technology, and the quantitative characterization of porosity is a crucial part of the research on coating structures. The current image analysis method often uses the mechanical polishing method recommended by ISO to measure a coating porosity. This method has been proved to be an effective method for the characterization of oxide coatings. However, due to the significant differences in the physical and chemical properties between aluminum and oxides, this method may not be suitable for aluminum coatings, and a more appropriate approach needs to be explored. In this paper, the effects of three polishing technologies (mechanical polishing, argon-ion-beam polishing, and electrolytic polishing) on the porosity measurement of pure aluminum coatings were compared and studied. The research results showed that the commonly used mechanical polishing method and more advanced argon-ion-beam polishing method could not completely reveal the pore structure because SiC particles would be embedded in the pure aluminum coatings during mechanical polishing, filling large pores. Although electrolytic polishing technology had advantages in revealing the macroporous structure, it would introduce a microporous structure and oxides, which would affect the measurement of the coating porosity. The composite polishing technology (electrolytic polishing + argon-ion-beam polishing) could perfectly reveal the pore structure in the pure-aluminum coating, and the porosity of arc-sprayed aluminum coating was 9.9%, which was close to the macroscopic true value measured using the weighing method of 10.2%.

## 1. Introduction

Thermal-spraying technology is a widely used surface treatment technology. It is being explored and developed for various applications, including those for which wear resistance, corrosion resistance and thermal protection are being sought [1,2,3]. However, the quantitative characterization of pores and the establishment of the relationship between the pores and the properties remain to be carried out before these coatings can be efficiently exploited and find widespread use [4,5,6]. Porosity is an inherent property of coatings. During the process of the preparation of thermal-spraying coatings, many factors, such as the impact energy of incomplete melted particles, the solidification and shrinkage of coating, the shielding effect caused by different spray angles, and the wide gap of the droplet’s velocity and temperature are accountable for the form of pore defects in the coatings [7,8,9]. The pore structure has an important influence on the service performance of coatings. A higher porosity can weaken some performances of the coating, such as the bond strength between the coating and the substrate, the cohesion strength between the coatings and the corrosion resistance of the coating [10,11]. Therefore, the quantitative characterization of the pores should be considered.

At present, many methods have been in development for the observation and measurement of the coating pores [12,13,14], including the image analysis method, mercury porosimetry, BET adsorption measurements, densitometry, high-resolution scanning electron microscopy, helium pycnometry, atomic-force probes, the contact measurement method, the ray-three-dimensional-scanning imaging method, etc. The image analysis method, based on scanning electron microscope technology, is simple in operation and can quantitatively characterize the number, shape, size, distribution and other characteristics of pores accurately. It has been proved to be a reliable pore-analysis method [15]. This method has also been recommended by the International Organization for Standardization (ISO 26946) as the standard method for evaluating the pores of thermal-sprayed coatings [16]. The procedure involves cross-sectioning the coating sample, then polishing and obtaining suitable images for stereographic protocols. This method, although suffering from the disadvantage of being destructive, provides an indication of porosity. The reliability of the results is influenced by factors that include (i) metallographic preparation, (ii) the imaging technique of the sample, (iii) the differences in the coating structure in different cross sections and (iv) post-processing techniques of the image, such as thresholding procedures and other technical details. At present, this method is widely used for the quantitative analysis of coating pores [17,18].

A thermal-sprayed aluminum coating has excellent corrosion resistance and a long service life, and is widely used for the surface protection of ordinary carbon steel [19,20,21]. According to the reported results, the porosity of the thermal-sprayed coating was between 10–20%. However, the results of porosity statistics obtained using the common image analysis method are often lower than the true value. The observation of porosity using image analysis strongly depends on the selection of the sample polishing process and the observation method [22]. It should be noted that the sample polishing process in the porosity analysis method of the coating recommended by ISO 26946 is applicable to oxide coatings, and whether it is also applicable to pure metal coatings should be carefully considered [23,24]. Therefore, this paper will compare the effects of different polishing processes on the quantitative characterization results of the pores of aluminum coatings, aiming to obtain more accurate quantitative characterization results for the pores of the coatings.

## 2. Experimental Process

### 2.1. Thermal Spraying

A commercially pure aluminum coating was prepared on an SS400 steel substrate using twin-wire arc-spraying technology. The substrate size was 150 mm × 100 mm × 5 mm, and the commercially pure aluminum wire diameter was 1.6 mm. The compositions of the SS400 steel and the commercially pure aluminum wire (AA1060) are shown in Table 1. The thermal-spraying process was completed using an Arcspray145 arc spraying system from Metallization Company. The movement of the spray gun was precisely controlled using a KUKA automatic robot. Prior to the deposition, the surfaces of the substrates were grit-blasted by white aluminum oxide to enhance the adhesion of the coating by means of mechanical interlocking mechanisms. The specific spraying parameters are shown in Table 2. The thickness of the commercially pure aluminum coating obtained under this spraying parameter was about 650 ± 50 μm.

### 2.2. Sample Preparation

The cross section of the coating sample was obtained using wire cutting, and the coating cross section was treated using mechanical polishing, argon-ion-beam polishing, and electrolytic polishing, respectively. 

The mechanical polishing process was carried out according to the ISO TR 26946-2011 standard. First, the sample section was ground with 240~2400 # SiC waterproof abrasive paper in turn, then polished with colloidal silica for 30 min, and finally, the sample was cleaned with ultrasonic for 5 min. 

**Figure 1:**
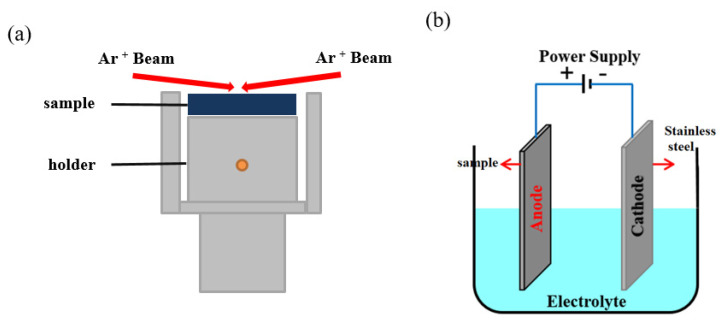
Schematic diagrams of (**a**) the argon-ion-beam polishing and (**b**) the electrolytic polishing.

The argon-ion-beam polishing of the coating section was carried out using a Gatan (Pleasanton, CA, United States) 679 polishing machine (Figure 1a), and the polishing parameters were 7 kV for 15 min, and then 4 kV for 10 min with last step at 1 kV for 10 min. The polishing angle was 2° and the stage rotation speed was 3 rpm during the polishing. 

The electrolytic polishing process was carried out on the self-made electrolytic device, and the schematic diagram of the device is shown in Figure 1b. The stainless steel was the cathode; the coating sample was the anode. The electrolyte was ethanol (AR 95%, Shanghai Boer Chemical, Shanghai, China) and perchloric acid (AR 70%, Shanghai Aladdin Chemical, Shanghai, China) with a volume ratio of 9 to 1, a polishing voltage of 20 V, and a time of 5–30 s. All polishing processes were carried out at room temperature.

### 2.3. Sample Characterization

The coating section was characterized using a field emission scanning electron microscope (FESEM, JEOL JSM-7800F, Tokyo, Japan) operated at 15 kV, and equipped with an X-ray energy spectrometer (EDS, Oxford Instrument MaxN) for the elemental analysis. Image J (version 1.53t) image processing software was applied to record statistics on the porosity of coatings. To remove possible noises from images, a contrast filter was implemented continuously. The image processing was eventually completed using image segmentation and filtering. Image segmentation was performed by thresholding leading to binary images and the isolation of pores of all types [8]. Eight SEM images were used to evaluate the porosities of the coating. 

## 3. Results

### 3.1. Mechanical Polishing

**Figure 2:**
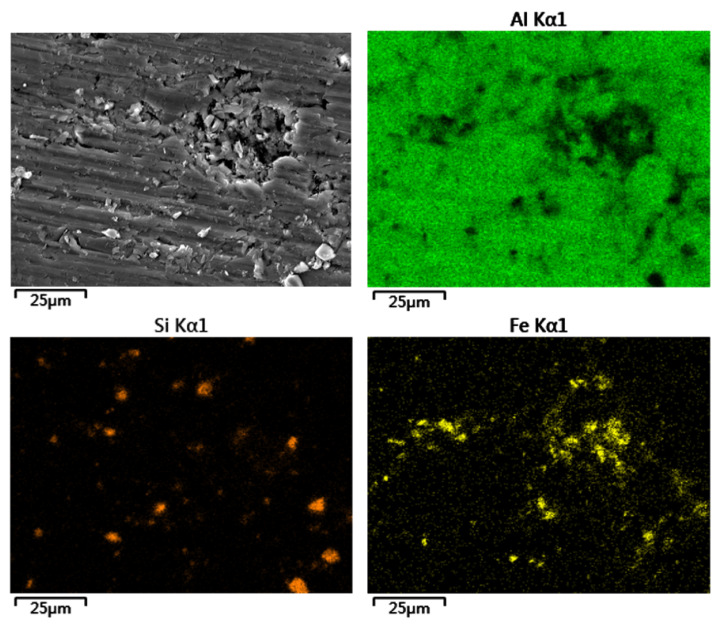
Surface morphology of the commercially pure aluminum coating after being mechanically ground and the resultant surface distribution of the corresponding elements.

The mechanically ground surface morphology of the coating is shown in Figure 2. After being ground by 240–2400 # SiC waterproof sandpaper in turn, the surface of the commercially pure aluminum coating had obvious scratches, which covered most of the pores; in particular, the large pores could hardly be seen. Through EDS element surface scanning of the coating section, as shown in Figure 2, it was found that a large amount of Si and Fe elements were concentrated on the commercially pure aluminum coating. These Si and Fe elements were the abrasive (SiC) and wear debris (SS400) in the grinding process that filled the coating pores. In addition, because the abrasive and abrasive chips were hard materials, they would also become embedded in a soft commercially pure aluminum coating during the grinding process.

**Figure 3:**
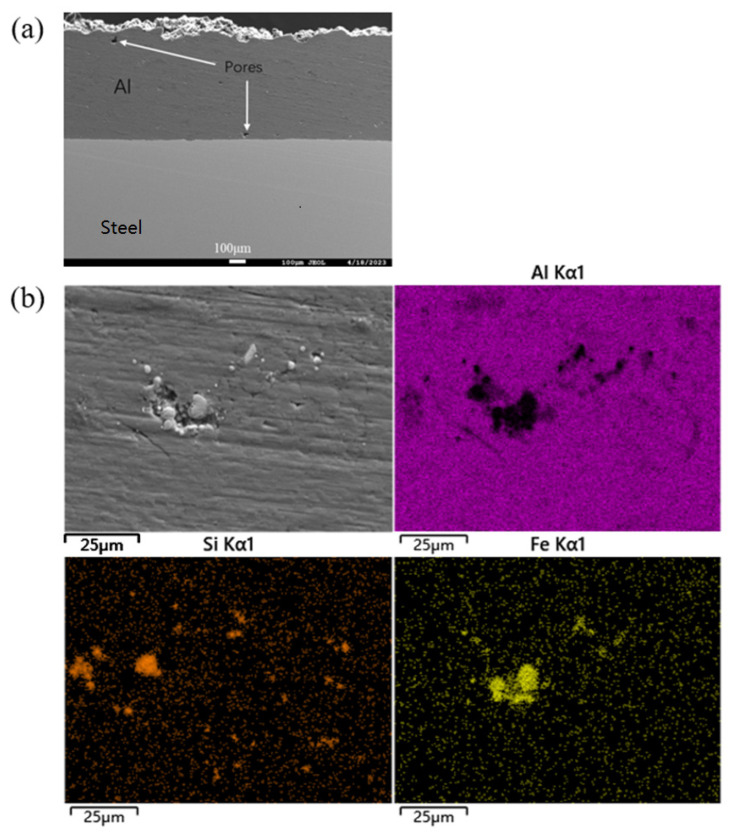
Surface morphology of the commercially pure aluminum coating after fine polishing and the surface distribution of the corresponding elements.

Through further fine polishing, the deep scratches in the coating could be removed, showing a certain pore structure, as shown in Figure 3a. According to the element distribution diagram, it could also be seen that the fine polishing partially removed the abrasives and wear debris in the coating, as shown in Figure 3b. However, the abrasive residue could not be completely removed because it was embedded too deep in the large pores, which would lead to large errors in the observation of large pores in the coating. The above results showed that the pore measurement method recommended by the current ISO 26946 could not be used to accurately measure the pores of the commercially pure aluminum coatings, and the mechanical polishing technology was not suitable to reveal the pore structure of metal coatings containing large pores.

### 3.2. Argon-Ion-Beam Polishing

**Figure 4:**
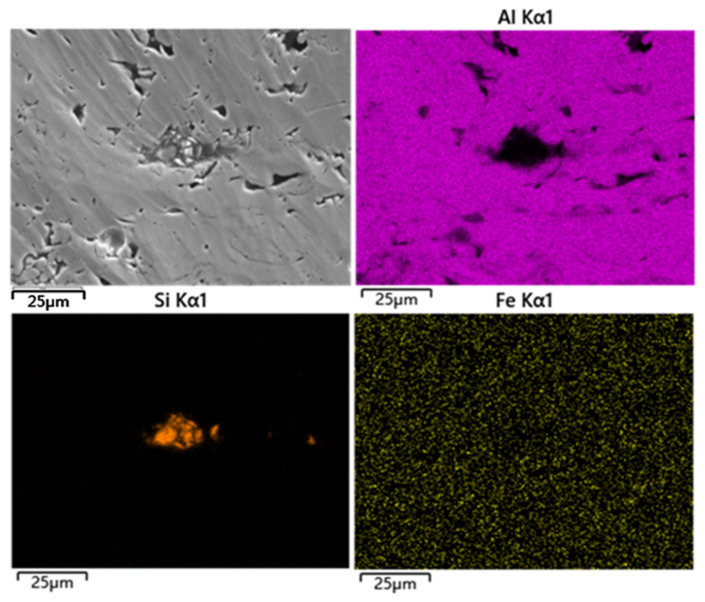
Surface morphology of commercially pure aluminum coating after argon-ion-beam polishing and the surface distribution of the corresponding elements.

Argon-ion-beam polishing technology is an advanced polishing technology at present, which can effectively remove the deformed layers, pollutants and residual stress on the surfaces of materials. It has been more-and-more-widely used in the surface treatment of SEM samples [25]. Figure 4 shows the surface morphology of a commercially pure aluminum coating after argon-ion-beam polishing and the corresponding surface element distribution. After high-energy argon ion beams treated the coating section, the scratches on its surface could be completely removed. After the polishing, the coating clearly showed the pore structure, especially the pores and interlayer cracks. It could be seen from the element plane distribution diagram that argon-ion-beam polishing completely removed the wear debris and most of the abrasives on the coating surface. However, further analysis found that there was still a small amount of the element Si in the coating, namely abrasive residue. These abrasive residues were often embedded in the large-size (25–100 μm) pores. The argon-ion-beam bombarded the coating surface at a small angle with limited depth of action, usually <30 μm, depending on the polishing parameters (angle, voltage, time), so it was difficult to completely remove the abrasives in the large pores.

As an alternative polishing method, the argon-ion-beam cross-section polishing technology could blast the thicker coating surface to obtain a pore structure with a depth of more than 100 μm [26]. However, the existing cross-section polishing technology had a low polishing efficiency, took a long time, and the available effective polishing area was very small, often less than 1 mm^2^, which caused a lack of data for the quantitative analysis of the pores. Therefore, the cross-section polishing method is not recommended.

### 3.3. Electropolishing

**Figure 5:**
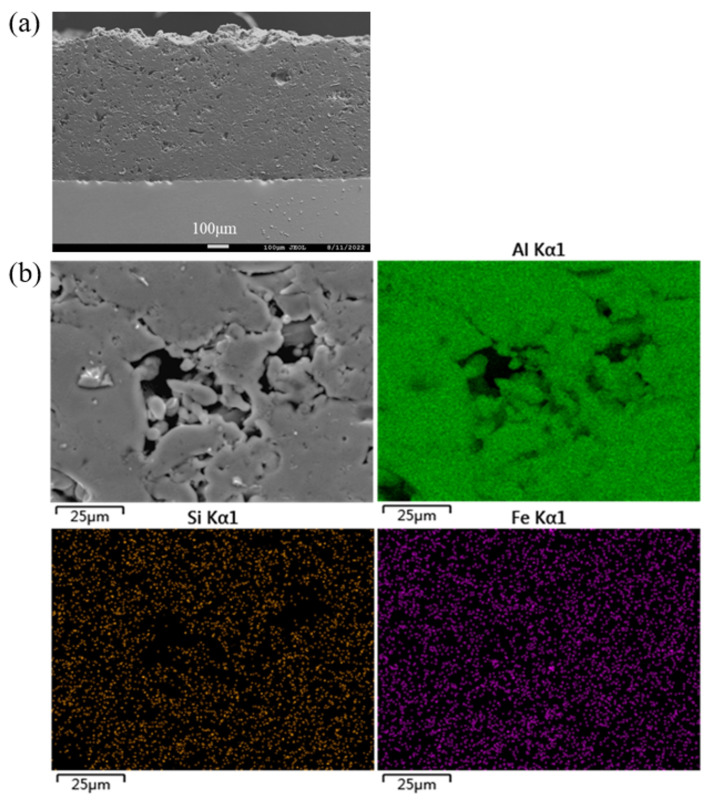
Surface morphology of commercially pure aluminum coating after electrolytic polishing (**a**) and enlarged surface morphology, and the corresponding surface distribution of the elements (**b**).

Electrolytic polishing, also known as electrochemical polishing, is a common surface treatment technology for metal materials, which can help to obtain smooth and bright surfaces [27,28]. As shown in Figure 5a, after electrochemical polishing, the scratch on the surface of the commercially pure aluminum coating was completely removed, and the pores of the various forms of the coating could be well displayed, in which the size of macropores exceeded 100 μm. As shown in Figure 5b, the element plane-distribution diagram did not contain the accumulation of Si and Fe elements. Electrolytic polishing completely removed the wear debris and abrasive residues in the coating. The above results showed that compared with the previous two polishing technologies, electrolytic polishing was more suitable for presenting the pore structure of the commercially pure aluminum coating. Early researchers also used similar chemical corrosion techniques to observe the pore structure of aluminum coating [29].

**Figure 6:**
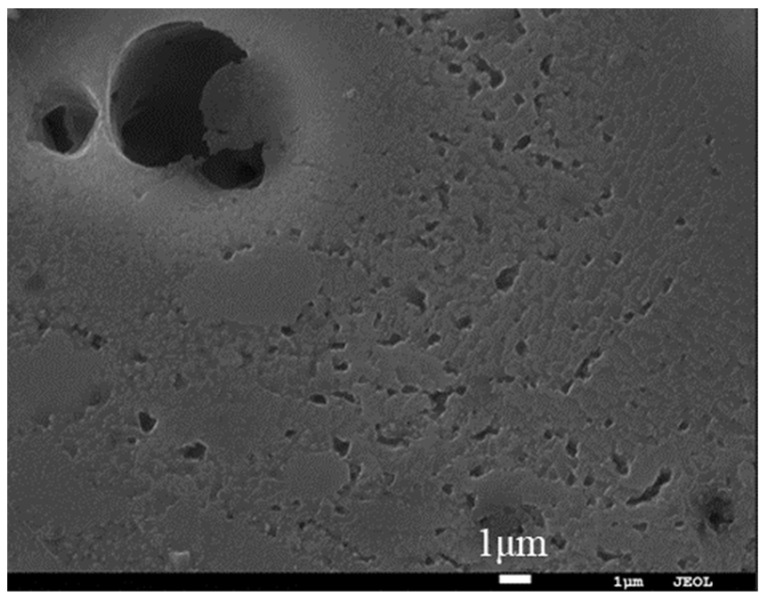
Enlarged surface morphology of commercially pure aluminum coating after electrolytic polishing.

The electrolytic polishing was essentially an anodic oxidation process. The commercially pure aluminum coating, as an anode, would generate a layer of oxide film on the surface when the oxidation reaction occurred. Under the action of an electric field, this oxide film would be dissolved in acid electrolyte, which would remove the wear debris and abrasive in the coating surface and display the pores at the same time. However, the anodic oxidation process of commercially pure aluminum often produced submicron-sized micropores on the surface. These pores were similar in size to the previous pores of the coatings, as shown in Figure 6. Under the SEM, the two kinds of micropores were often indistinguishable, which affected the statistical analysis of the coated micropores.

Table 3 shows the porosity results of the commercially pure aluminum coating obtained after three polishing processes. By comparing the above three results, it can be seen that the commonly used mechanical polishing technology and the more advanced argon-ion polishing technology had great difficulties in characterizing the large pores of the commercially pure aluminum coating, resulting in the porosity statistics often being less than the true value measured using the weighing method of 10.2%, which was below (0.5 ± 0.3)% and (5.0 ± 0.8)%, respectively. In contrast, the electrolytic polishing technology showed advantages in characterizing macropores, and the porosity it obtained was (8.6 ± 1.8)%, which was close to the macroscopic true value measured using the weighing method of 10.2%. This technology is, therefore, worth popularizing in practical measurement methods. However, as previously mentioned, it is difficult to reveal small pores using electrolytic polishing. In conclusion, the current single polishing technology cannot fully reveal the pore structure of commercially pure aluminum coating.

## 4. Discussion

When high-speed molten particles hit the formed coating surface, the pores will inevitably be generated in the coating due to the incomplete filling of the rough surface and its incomplete combination with particles. The pores in the coating are generally composed of large pores of several microns to tens of microns, and small pores on the submicron scale [6]. Porosity is one of the quantitative indicators that reflects the quality of a coating. On one hand, holes can improve the wear resistance of wear-resistant coatings when holes are used to store abrasives; on the other hand, corrosive substances can penetrate holes and come into contact with the surface of workpieces. The distribution, morphology and size of pores in the coating will affect the performance of the coating, and there are many pores at the interface between the coating and the substrate, which will seriously weaken the bonding strength between the coating and the substrate [6]. At present, the accurate measurement of porosity is an important part of coating-structure characterization. For arc-spraying technology, the overall porosity of a coating is 10–20% [17].

At present, the mechanical polishing method recommended by ISO 26946 is effective for the quantitative characterization of oxide-coating pores, where the pore size is often less than 20 μm. The properties of commercially pure aluminum coating and oxide coating are greatly different. In particular, there are very large pores in the commercially pure aluminum coating, which can exceed 100 μm at most. This leads to hard SiC particles becoming easily embedded in the soft commercially pure aluminum coating or filling the pores during the polishing process. The main purpose of the later polishing process is to remove these SiC particles. Among the three polishing methods introduced here, mechanical polishing and the argon-ion-beam polishing cannot completely remove the SiC particles in the macropores, which leads to the quantitative statistical value of porosity being far less than the true value. Currently, most of the reported literature shows the pore structure of commercially pure aluminum coating treated using mechanical polishing, and the measured coating porosity is small as a whole. For example, by analyzing the pore structure of pure aluminum coating according to the SEM images of He et al., it can be seen that the porosity was less than 2%, while the real porosity obtained using the density weighing method reached 17.4% [30]. This result also proves that mechanical polishing is not suitable when measuring the porosity of an aluminum coating using image analysis method.

**Figure 7:**
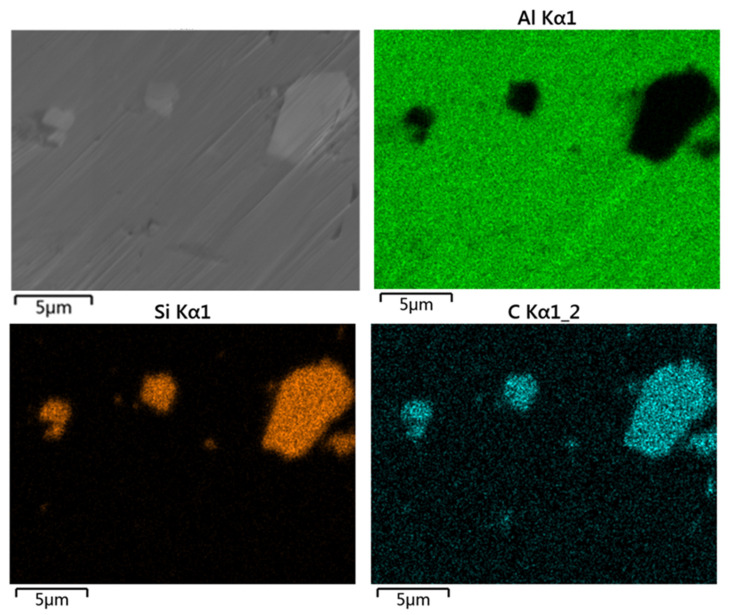
Surface morphology of SiC particles in the commercially pure aluminum coating after argon-ion-beam polishing and the corresponding distribution of elements surface.

As a more advanced polishing technology, the argon-ion-beam polishing can completely remove surface scratches and obtain structural information such as submicron small pores and interlayer defects, but with this method it is hard to remove SiC particles completely in the macropores due to its insufficient polishing efficiency and depth of action. Figure 7 shows more evidence that SiC particles with different sizes are embedded tightly in the coating after polishing with an argon ion beam. These particles, under, SEM have a similar image contrast to the coatings, leading to a smaller statistical value of porosity. In contrast, electrolytic polishing have certain advantages towards the porosity of commercially pure aluminum coating. It can effectively remove SiC particles in the pores, showing a macroporous structure, and the porosity statistics obtained are close to the true value. However, the microporous structure produced by electropolishing is a problem that cannot be ignored [31,32]. Although in the process of image processing, the influence of these micropores on the porosity statistics of the coating is deleted by setting a threshold value, if there are a large number of micropores in the coating, a large error will be caused in the porosity calculation.

**Figure 8:**
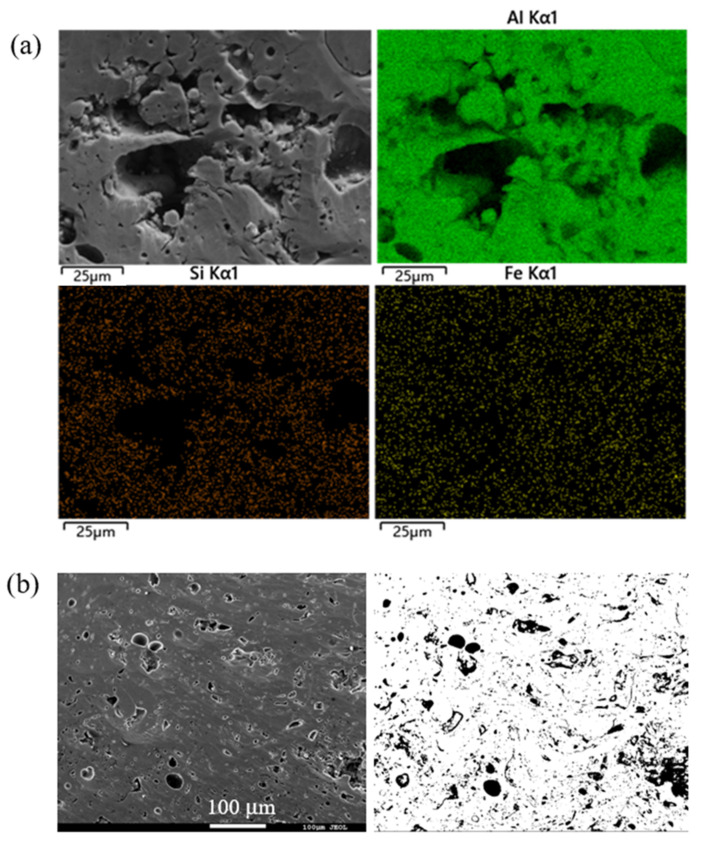
Surface morphology of the commercially pure aluminum coating after the composite polishing method and the corresponding distribution of surface elements (**a**), and the SEM image used for pore statistical analysis (**b**).

For this reason, a composite polishing technology was proposed, that was the combination of electrolytic polishing and argon-ion-beam polishing. For the polished coating samples, firstly, it took the advantages of electrolytic polishing to remove the SiC particles in the coating, showing the large pore structure, and then applied the argon-ion-beam polishing technology to remove the micro pore structure introduced in the electrolytic polishing process, so that the real pore structure of the coating was finally obtained. A typical result is shown in Figure 8a. With this composite polishing technology, the abrasives and wear debris could be removed completely, and the pore structures in the coating are well displayed in the SEM image. After statistical analysis, the number, shape, distribution and other information of the pores of different sizes could be obtained using the image analysis method (Figure 8b). The relevant statistical results are shown in Table 4. Finally, the porosity was (9.9 ± 0.8)%, which was very close to the true value.

If we look back to the results of Table 3 and compare the figures (Figure 2, Figure 3a, Figure 5a and Figure 8b) of the four types of polishing processes at relatively low magnifications, advantages and disadvantages can be seen regarding the four polishing methods of the commercially pure aluminum coating: (1) current mechanical polishing is not suitable for porosity measurements of commercially pure aluminum coating; (2) argon-ion-beam polishing has the advantage of displaying the pore structures, but it is time-consuming and only affects small areas; (3) electrolytic polishing is effective in revealing the big pores of commercially pure aluminum coating, but it causes micropores on the coatings; and (4) the composite polishing, which can not only reveal big pores but also remove micropores, is considered to be an ideal method for porosity measurements of a commercially pure aluminum coating. 

## 5. Conclusions

(1) Current mechanical polishing and advanced argon-ion-beam polishing are not suitable for revealing the pore structure of a commercially pure aluminum coating, and lead to less porosity statistics.
(2) The electropolishing method can effectively reveal the macropore structure of a commercially pure aluminum coating, but the influence of introduced micropores on its porosity statistics cannot be ignored.
(3) The composite polishing technology (electrolytic polishing + argon-ion-beam polishing) can truly reflect the pore structure of a commercially pure aluminum coating, and the porosity of the coating obtained is 9.9%.
(4) Using the composite polishing technology, electrolytic polishing for 15 s and argon-ion-beam polishing for 35 min (7 kV for 15 min, and then 4 kV for 10 min, with a last step at 1 kV for 10 min) is recommended to reveal the porosity of a commercially pure Al coating.

## Figures and Tables

**Table 1:** Compositions of the SS400 steel and the commercially pure aluminum wire (in mass %).

	Mg	Si	Cu	Mn	Zn	Ti	S	P	C	Fe	Al
SS400	-	0.30	-	1.20	-	-	0.03	0.04	0.20	balance	
AA1060	0.03	0.25	0.05	0.03	0.05	0.03	-	-	-	0.35	balance

**Table 2:** Process parameters used for twin-wire arc spray.

Parameters	Values
Voltage, V	150
Current, A	30
Distance, mm	180
Speed, m/s	0.05
Coating thickness, μm	600–700

**Table 3:** Porosities of commercially pure aluminum coating after different polishing processes.

Polish Method	Mechanical Polishing	Argon-Ion-Beam Polishing	Electrolytic Polishing	Combination Polishing
Porosity (%)	<0.5 **± 0.3**	<5.0 **± 0.8**	~8.6 **± 1.8**	~9.9 **± 0.8**

**Table 4:** Pore information from Figure 8b.

Pore Character	Shape	Size (μm)	Number	Distribution
Interconnected pore	Irregular	25–100	6	Inhomogeneous
Isolated pore	Round or irregular	2–20	41	Inhomogeneous
Interlayer pore	Curved	10–100 (in length)	18	Inhomogeneous
Micro pore	Round	<1	286	Uniform

## Data Availability

The study did not report any data.
